# A Chronic Ocular-Hypertensive Rat Model induced by Injection of the Sclerosant Agent Polidocanol in the Aqueous Humor Outflow Pathway

**DOI:** 10.3390/ijms20133209

**Published:** 2019-06-29

**Authors:** Román Blanco, Gema Martinez-Navarrete, Consuelo Pérez-Rico, Francisco J. Valiente-Soriano, Marcelino Avilés-Trigueros, Javier Vicente, Eduardo Fernandez, Manuel Vidal-Sanz, Pedro de la Villa

**Affiliations:** 1 Department of Surgery, Medical and Social Sciences, University of Alcalá, Alcalá de Henares, 28805 Madrid, Spain; 2 Instituto Ramón y Cajal de Investigación Sanitaria (IRYCIS), 28034 Madrid, Spain; 3 Institute of Bioengineering, Miguel Hernandez University, Elche, 03202 Alicante, Spain; 4 Biomedical Research Networking consortium in Bioengineering, Biomaterials and Nanomedicine (CIBER-BBN), 28029 Madrid, Spain; 5 Department of Ophthalmology, University Hospital Principe de Asturias, Alcalá de Henares, 28805 Madrid, Spain; 6 Department of Ophthalmology, University of Murcia and Institute Murciano of Investigation Biosanitaria-University Hospital Virgen de la Arrixaca (IMIB-Arrixaca), 30120 Murcia, Spain; 7 Universidad Europea de Madrid, Villaviciosa de Odón, 28670 Madrid, Spain; 8 Department of Systems Biology, University of Alcalá, Alcalá de Henares, 28805 Madrid, Spain

**Keywords:** chronic ocular hypertension, optical coherence tomography, pattern and full-field electroretinogram, polidocanol injection, rat glaucoma model

## Abstract

Background: To induce a moderate chronic ocular hypertension (OHT) by injecting polidocanol, a foamed sclerosant drug, in the aqueous humor outflow pathway. Methods: Intraocular pressure (IOP) was monitored for up to 6 months. Pattern and full-field electroretinogram (PERG and ERG) were recorded and retinal ganglion cells (RGC) and retinal nerve fiber layer (RNFL) thickness were assessed in vivo with optical coherence tomography (OCT) and ex vivo using Brn3a immunohistochemistry. Results: In the first 3 weeks post-injection, a significant IOP elevation was observed in the treated eyes (18.47 ± 3.36 mmHg) when compared with the control fellow eyes (12.52 ± 2.84 mmHg) (*p* < 0.05). At 8 weeks, 65% (11/17) of intervention eyes had developed an IOP increase >25% over the baseline. PERG responses were seen to be significantly reduced in the hypertensive eyes (2.25 ± 0.24 µV) compared to control eyes (1.44 ± 0.19 µV) (*p* < 0.01) at week 3, whereas the ERG components (photoreceptor a-wave and bipolar cell b-wave) remained unaltered. By week 24, RNFL thinning and cell loss in the ganglion cell layer was first detected (2/13, 15.3%) as assessed by OCT and light microscopy. Conclusions: This novel OHT rat model, with moderate levels of chronically elevated IOP, and abnormal PERG shows selective functional impairment of RGC.

## 1. Introduction

Glaucoma is a major cause of irreversible blindness worldwide and is characterized by progressive optic nerve and retinal ganglion cells (RGC) degeneration [1]. Because high intraocular pressure (IOP) is an important risk factor associated with glaucoma, several animal models of elevated IOP have been developed in multiple species, including rabbits, dogs, nonhuman primates, rats, and mice with the objective of improving our understanding of glaucoma and its treatment [2].

Most animal models of chronic glaucoma are based mainly on elevation of the IOP leading to the characteristic optic neuropathy with degeneration of RGCs. Rodent glaucoma models have been summarized recently and although the anatomy of the human eye is different from the rat optic nerve head, the ultrastructural relationships between axons and glia are similar, making it likely that cellular axonal degeneration processes in these models will be relevant to human glaucoma [3].

Induced ocular hypertension (OHT) models include laser photocoagulation of the trabecular meshwork [4,5], injection of hypertonic saline into episcleral veins [6], injection of substances into the anterior chamber to block aqueous outflow, laser-induced anterior synechiae [7], and cauterization of episcleral veins [8]. All these hypertensive methods vary according to the dynamics and peak level of the IOP elevation. All these methods share the main drawbacks described by Bierman et al. [7] e.g., acute peaks, slow rise of IOP, or early normalization of IOP levels before significant RGC damage occurs, needing re-treatment and variable IOP increments during follow-up treatment. Among the techniques that include a ‘vascular approach’ to increasing IOP, the injection of hypertonic saline into episcleral veins and cauterization of episcleral veins are the most popular [6,8]. The etiopathogenic mechanism proposed for the first technique is the toxicity of the hypertonic saline to the trabecular meshwork, as shown by histologic data [6]. In the present study, we used a substance, polidocanol, to alter the aqueous humor drainage pathway. Polidocanol is a sclerosant agent used in clinics for treating venous disorders and vascular malformations [9]. Polidocanol is included in the surfactant category among the sclerosant agents, and its mechanism of action is concentration-dependent, mainly causing endothelial cell death but potentially can involve platelet and erythrocyte death [10]. As the whole drainage system from the trabecular meshwork to the episcleral veins is a modified vascular system, we started with the hypothesis that injection of polidocanol could interfere with the whole aqueous humor collector system. We assumed that a more extensive damage to the drainage system might generate an increased IOP level that persisted for longer periods than in previous models.

Although the factors provoking ultimate RGCs death stand disputed, increasing evidence from experimental animal models and human patients shows that glaucoma displays an initially moderate and diffuse loss of inner retinal responses [11]. Consequently, lower levels of chronically elevated IOP in rodent models of experimental glaucoma [3] may be more applicable to human OHT and primary glaucoma. There is evidence that even IOP only slightly above normal is capable of producing RGC dysfunction [11,12] and that only small IOP elevations are needed for animal models to be used more effectively to test new neuroprotective and anti-glaucoma compounds [13,14]. 

In this study, we describe a method of inducing a moderate and persistent elevated IOP by injecting polidocanol, a foamed sclerosant drug, in the aqueous humor outflow pathway in order to overcome the drawbacks of the other experimental techniques previously mentioned. We functionally evaluated this model for its selectivity in impairing RGCs by examining the retinal function of RGCs and photoreceptor-mediated responses using the pattern and full-field electroretinogram (PERG and ERG). We also evaluated retinal nerve fiber layer (RNFL) thickness by using the spectral-domain optical coherence tomography (OCT) and counted the total population of RGCs using Brn3a immunohistochemistry.

## 2. Materials and Methods

### 2.1. Animal Handling and Ethics Statement

This study was conducted in accordance with the Association for Research in Vision and Ophthalmology Guide for the Care and Use of Laboratory Animals and all protocols used were authorized by the animal studies and ethical committee of the University of Alcalá (RD16/0008; 01/01/2017). Adult male albino Wistar rats (8–10 weeks old, 180–230 g) were housed in constant light- (about 75 lux) and temperature-controlled conditions with freely available food and water. Animal surgery and IOP measurements were performed under anesthesia with isoflurane/O_2_. ERG recordings and OCT measurements were performed under anesthesia with i.p. ketamine (40 mg/kg, Imagene^®^; Merial, Madrid, Spain) and xylazine (5 mg/kg, Rompun^®^; Bayer, Madrid, Spain). 

### 2.2. Experimental Design

In all rats, the right eyes were used as experimental specimens and the left eyes served as untreated controls. Only those rats showing IOP elevation in the first ten days after polidocanol injection in the perilimbal plexus were selected for further longitudinal monitoring. IOP was measured in both eyes, starting one day prior to intervention and repeated every other day for the first week post-injection and then every week. PERG recordings were taken in both eyes, one day prior to injection and 3 weeks after polidocanol injection. Full-field ERG responses were also recorded simultaneously from both eyes at 3 weeks post-surgery. Non-invasive in vivo assessment of eye structures was performed using the Spectralis OCT system at 2, 12 and 24 weeks. After ERG and OCT measurements were taken, a first batch of rats was euthanized at 12 weeks (*n* = 4) and a second group at 24 weeks (*n* = 13) for Brn3a Immunohistochemistry. A separate batch of hypertensive and control eyes (*n* = 3 per group) were used to study morphologic anterior segment changes at two weeks.

### 2.3. Surgical Technique

**Figure 1:**
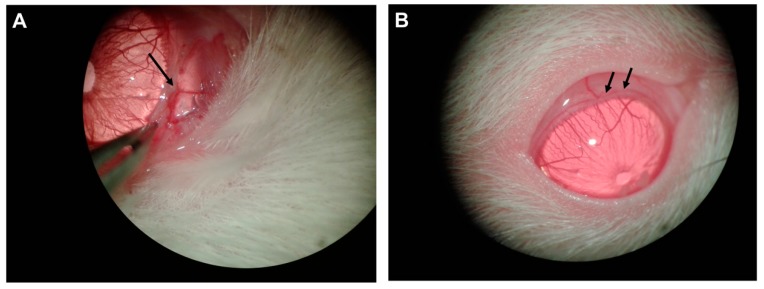
Induction of chronic intraocular pressure (IOP) elevation. (**A**) The limbal vascular plexus was exposed after carefully dissecting the conjunctiva. Then, a microneedle was positioned tangentially to the cornea and a volume of 50 µL polidocanol 3% was slowly injected into the perilimbal vessels. (**B**) Vessel blanching (arrows) and the extent to which the sclerosant went through the limbal vessels were carefully evaluated after injection.

The microneedle injection technique used is similar to the one described elsewhere [6]. Under a surgical microscope, the eye was positioned to expose a circumferential vein of the limbal plexus in the upper quadrant. The conjunctiva and Tenon’s capsule were dissected to expose the vein wall. Then, a glass microneedle was positioned parallel to the vessel axis, inserted into the vessel lumen (Figure 1) and a volume of 50 μL of polidocanol (Etoxiesclerol 3%, Ferrer Farma, Barcelona, Spain) dissolved in distilled water was slowly injected.

### 2.4. Intraocular Pressure Measurements

IOP was measured in both eyes with a rebound tonometer (Tonolab, Icare, Vantaa, Finland), starting one day prior to intervention and repeated every other day for the first ten days post-injection and then every week. To avoid the effects of diurnal fluctuations, IOP was measured at the same time of the day (between 10 am and 12 pm) and under similar lightning settings [15,16,17,18,19]. To minimize the time-dependent effects of isoflurane/02 anesthesia, IOP readings were obtained within three minutes after induction of anesthesia [17]. To compare IOP measurements obtained from both eyes, IOP readings were randomly obtained from the injected or control eye. The IOP for each eye was defined as the average of ten measurements recorded for each eye. Only those rats showing IOP elevation in the first ten days after polidocanol injection (17/25, 68%) were selected for further longitudinal monitoring. Slit-lamp biomicroscopy was performed in the first day’s post-surgery to look for any signs of anterior chamber inflammation.

### 2.5. Pattern Electroretinogram (PERG)

PERG recordings were taken simultaneously in both eyes, one day prior to injection and 3 weeks after polidocanol injection. The animals were dark-adapted for 10 min prior to the start of the recording [11,20]. A Burian–Allen bipolar contact lens electrode (Hansen Labs, Coralville, IA, USA) was used. The reference and ground electrodes were placed on the forehead and the tail, respectively. Electrical impedance was always below 3 kΩ. The visual stimulus was generated by commercially available software (RETIcom; Ronald Consult, Brabdenburg, Germany). The stimulus consisted of light and dark (8 × 8) checkerboards that alternated at a frequency of 3 Hz with a 50% duty cycle. Display mean luminance was 100 cd-s/m^2^ and contrast was kept at 99%. Stimulus spatial frequency was 310 cpd and sweep time was 300 ms. Signals were amplified with a 2–250 Hz bandpass and digitized at a rate of 5 KHz [21,22]. 

### 2.6. Full-Field Electroretinogram (ERG)

Full-field ERG responses were recorded simultaneously from both eyes at 3 weeks post-surgery, as previously described [23,24]. Animals were dark-adapted overnight (>12 h) and all set-up preparations were done under dim red light. Before taking ERG recordings, pupil mydriasis was provoked with tropicamide 1% (AlconCusí, Barcelona, Spain). The recording system was similar to the one used for PERG. A Ganzfeld dome was used for light stimulation, which provided consistent illumination. Single-flash scotopic ERGs were recorded for intensities above 3.30 log cd-s/m^2^. After 15 minutes of light adaptation (150 cd/m^2^), photopic flash responses were recorded for light intensities between 0.97 and 2.72 log cd-s/m^2^ in 0.25 log unit increments. Each recording was an average of at least 20 responses with a two-second interstimulus interval. ISCEV (International society for clinical electrophysiology of vision) standard protocols were followed to measure waves amplitudes and implicit time responses [25].

### 2.7. Spectral-Domain Optical Coherence Tomography (OCT) 

Non-invasive in vivo assessment of eye structures was performed using the Spectralis OCT system (Heidelberg Engineering, Heidelberg, Germany) at survival intervals of 2, 12 and 24 weeks. A custom-made permeable contact lens (focal length: 10 mm, +25 D) was used as a collimator and to reduce the risk of edema and corneal dehydration. A horizontal B-scan centered on the optic nerve was performed, consisting of 1024 A-scans, with lateral and axial-resolution of 2 μm. Ten B-scans were repeated and averaged. ImageJ software (National Institutes of Health, Bethesda, MD) was used for image analysis. Retinal sublayer measurements consisted of the outer nuclear layer (ONL), inner nuclear layer (INL) and retinal nerve fiber (RNFL)/ganglion cell layer (GCL) thickness [26,27].

### 2.8. Brn3a Immunohistochemistry

After ERG and OCT measurements were taken, a first group of rats was euthanized at 12 weeks (*n* = 4) and a second group at 24 weeks (*n* = 13). The rats were transcardially perfused with 4% paraformaldehyde in phosphate buffered saline and both retinas were cut as whole-mounts and subjected to Brn3a immunofluorescence (goat anti-Brn3a diluted at 1:500; C-20; Santa Cruz Biotechnologies, Heidelberg, Germany), as reported elsewhere [2,28]. Secondary detection was performed using donkey anti-goat Alexa Fluor 568 diluted at 1:500 in blocking buffer (Molecular Probes; Invitrogen, Barcelona, Spain). Retinal whole-mounts were analyzed and photographed under an epifluorescence microscope, and Brn3a^+^ RGCs were automatically quantified, as reported elsewhere [27,28,29].

### 2.9. Histological Examination of the Anterior Segment 

A batch of hypertensive (IOP) and control eyes (IOP) were used to study morphologic anterior segment changes. Two weeks post-injection, IOP in the treated eyes was 19.67 ± 2.25 mmHg compared with the untreated fellow eyes (11.72 ± 2.84 mmHg) (mean ± SD) (t-test, *n* = 6, *p* < 0.05). Animals were killed at 2 weeks with an overdose of anesthetics and then perfused transcardially with 4% paraformaldehyde. Whole globes were formalin fixed and embedded in paraffin. Care was taken to ensure that the orientations of the eyes were identical by using tissue marking dye and cut into 3.5 μm sections. Deparaffinized tissues were stained with hematoxylin and eosin (HE) in 0.1% ethanol solution or periodic acid-Schiff reaction (PAS) following standard protocols. 

### 2.10. Data Analysis

For the statistical analysis, we performed repeated measures ANOVA and Student’s t-tests using commercial statistical analysis software (Prism 6; GraphPad Software Inc, La Jolla, CA, USA). We set significance at *p* < 0.05 for all analyses, and values are expressed as mean plus or minus SD. 

## 3. Results

IOP elevation was induced in the right eye of all the animals, while the left eye was used as an untreated reference. After sclerosing the episcleral vessels and limbal plexus, significant elevation of IOP was recorded in 17/25 (68%) of treated eyes. Only those rats that showed a consistent increase of IOP ten days after polidocanol injection were further monitored for up to 24 weeks. No signs of anterior chamber inflammation were observed under slit-lamp biomicroscopy in the first ten days post-polidocanol injection.

**Figure 2:**
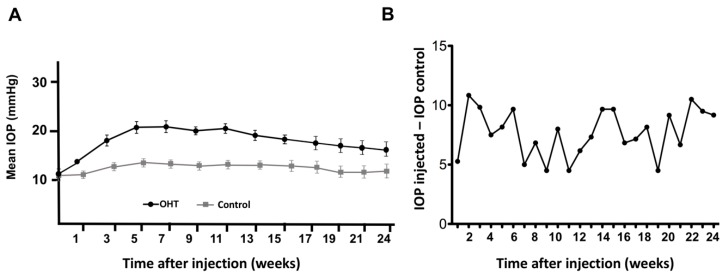
IOP values shown at baseline and up to week 24. (**A**) Maximum IOP elevation in the treated eyes was sustained and declined slowly over time. At 24 weeks, there were still significant differences between IOP intervention eyes. (**B**) IOP difference (IOP intervention - IOP control) for one illustrative animal monitored for 24 weeks.

There were no differences between the baseline IOP intervention eyes (11.12 ± 3.26 mmHg) (mean ± SD) and the baseline and subsequent IOP measurements of the control eyes (11.74 ± 3.17 mmHg) (mean ± SD) (repeated measures ANOVA, *n* = 17, *p* > 0.05). IOP increased steadily and no significant pressure spikes were observed in the first days post-injection. Three weeks post-injection, IOP in the treated eyes was 18.47 ± 3.36 mmHg compared with the control fellow eyes (12.52 ± 2.84 mmHg) (repeated measures ANOVA, *n* = 17, *p* < 0.05) and this significant IOP difference was sustained over time (repeated measures ANOVA, *p* < 0.01), as shown in Figure 2A. IOP difference (IOP intervention - IOP control) for one illustrative animal monitored for 24 weeks is shown in Figure 2B. The overall average IOP in the treated eye was 19.05 ± 0.75 mmHg (mean ± SD) compared to 12.02 ± 0.67 mmHg in the control fellow eye (*p* < 0.05) and the average IOP difference was 7.29 mmHg ± 0.44, showing a relatively large IOP variability during the study follow-up. 

At 12 weeks, there were significant differences between IOP intervention eyes (17.22 ± 2.68 mmHg) and IOP control eyes (11.99 ± 3.67 mmHg) (mean ± SD) (repeated measures ANOVA, *n* = 17, *p* < 0.05). 23% (4/17) of intervention eyes had developed an IOP increase >60% over the baseline at 12 weeks and 65% (11/17) had an IOP increase >25%. At 24 weeks, there were still significant differences between IOP intervention eyes (16.56 ± 3.95 mmHg) and IOP control eyes (11.37 ± 2.99 mmHg) (mean ± SD) (repeated measures ANOVA, *n* = 13, *p* < 0.05). 

**Figure 3:**
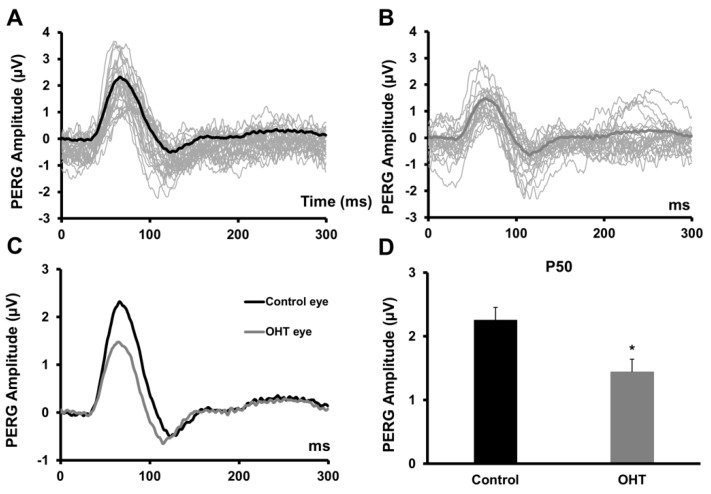
Effect of chronic IOP elevation on the retinal ganglion cells (RGCs) function. Pattern electroretinogram (PERG) P50 amplitude responses were significantly reduced in all hypertensive eyes. (**A**,**B**) Representative individual examples of PERG recordings of control (A) and ocular hypertension (OHT) (**B**) eyes. Grey traces represent the individual waveforms and the superimposed black trace represents the average PERG responses. (**C**) Average PERG waveform responses, showing the difference between the hypertensive (grey) and untreated fellow control eyes (black). The elevation of IOP caused a remarkable average reduction (36%) in retinal responsiveness to the stimulating pattern. (**D**) Bar graphs showing the PERG P50 amplitude results. A significant difference in the P50 amplitude was observed between hypertensive eyes and control fellow eyes (*p* < 0.01).

The repercussion of chronic IOP elevation on the RGCs function was measured with the PERG three weeks after the surgical procedure (Figure 3). At baseline, there was not any difference between the intervention eyes (2.37 ± 0.16 µV) (mean ± SD) and the control eyes (2.05 ± 0.12 µV) (mean ± SD) (t-test, *n* = 17, *p* > 0.05). At 3 weeks, the treated eyes’ response averages were significantly reduced (1.44 ± 0.19 µV, mean ± SD), while the mean responses in the control fellow eyes (dark bar) were 2.25 ± 0.24 µV (t-test, *n* = 17, *p*< 0.01). PERG responses in the hypertensive eyes were significantly diminished (t-test, *n* = 17, *p* < 0.05), being, on average 36% lower than the responses recorded in the reference fellow eyes. 

**Figure 4:**
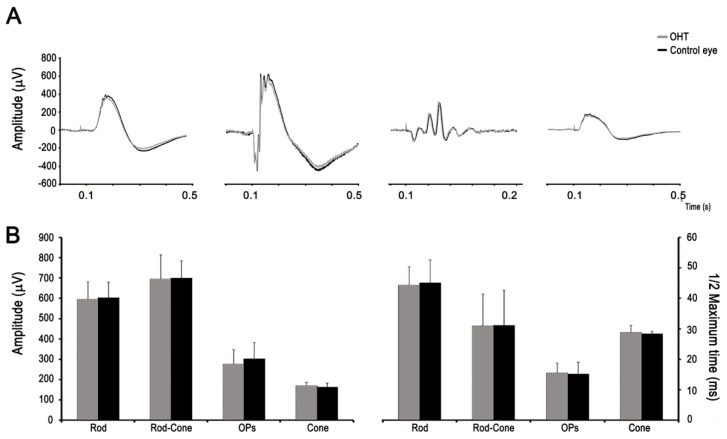
Effect of chronic IOP elevation on the outer retina function. (**A**) Normal scotopic (rod) and photopic (cone) full-field electroretinogram (ERG) recordings from a representative animal (hypertensive eye in gray traces and control eye in black) are shown. (**B**) Bar graphs showing the amplitude and implicit time responses for the dark-adapted (rod, combined rod-cone, and oscillatory potentials (OP)) and light-adapted (cone responses) ERG responses. There were no significant differences in the amplitudes and implicit times of the various ERG components between the hypertensive and control fellow eyes.

The effect of chronic IOP elevation on the outer retina function was also examined with the ERG at the same time as the PERG was performed (3 weeks). It is already known that changes in the response to brighter ERG flashes would be suggestive of effects on retinal cells (photoreceptors) other than RGCs. Our ERG recordings did not show any damage to the outer retina and photoreceptors and the ERG amplitudes and implicit times of the hypertensive eyes were not statistically different from those of the control fellow eyes (t-test, *p* > 0.05) (Figure 4), an important difference from other ocular hypertensive models in rodents [30]. The scotopic responses were 294.1 ± 90.11 µV in the hypertensive eyes and 288.06 ± 99.51 µV in the fellow eyes. Mixed rod-cone and photopic responses were 695.20 ± 169.32 µV in the hypertensive eyes and were 700.48 ± 117.73 µV in the control eyes. Photopic responses were 169.37 ± 27.80 µV in the hypertensive eyes and 162.38 ± 29.53 µV in the control eyes. These ERG results support the hypothesis that low-to-moderate levels of elevated IOP need to be achieved in rodent models of experimental glaucoma in order for them to be comparable to human chronic open-angle glaucoma [31]. 

**Figure 5:**
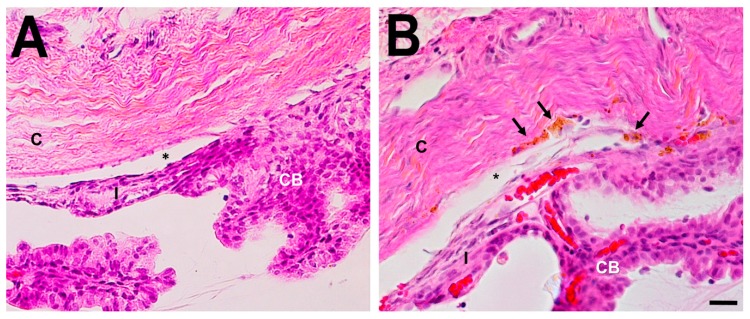
Histological examination of the anterior chamber angle two weeks after polidocanol injection. (**A**) Light microscopy of hematoxylin-eosin staining sections of the iridocorneal angle from an uninjected left control eye illustrating normal histology of trabecular meshwork area. (**B**) Angle from an experimental eye shows the presence of macrophages (arrows) on the trabecular meshwork area. Anterior chamber (*), cornea (C), ciliary body (CB) and iris root (I). Scale bar 20 μm.

Histologic analysis of the anterior segment consistently showed pigment containing macrophages present in the trabecular meshwork and loss of the normal trabecular meshwork architecture in the hypertensive eyes (Figure 5). Iridocorneal adhesions were not seen and the cornea, anterior chamber and the lens were clear and not affected macroscopically. Schlemm’s canal, the collector channels, and the veins of the limbal vascular plexus were congested, indicating that the main impediment to aqueous outflow was at the level of the trabecular meshwork. The ciliary process epithelium appeared normal. Histologic examination of control eyes revealed a normal anterior chamber angle and Schlemm’s canal. Histology of non-responsive eyes (not shown) looked like control eyes, so one may assume that the polidocanol did not reach the outflow tissues, being a technically challenging procedure.

Retinal microstructure was analyzed by means of the OCT. At both week 2 and week 12, we did not observe any significant reduction in RNFL + GCL + IPL thickness (73.13 ± 3.46 µm) in the hypertensive eyes compared to the control eyes (74.52 ± 0.62 µm) (repeated measures ANOVA, *n* = 17, *p* > 0.05). Moreover, whole retina (194.98 ± 10.93 µm), ONL (49.49 ± 4.08 µm) and INL thicknesses (17.23 ± 1.44 µm) in the hypertensive eyes were not significantly different from the fellow control eyes’ whole retina (191.40 ± 10.93 µm), ONL (51.72 ± 5.07 µm) and INL thickness values (18.21 ± 0.87 µm) (repeated measures ANOVA, *n* = 17, *p* > 0.05). 

**Figure 6:**
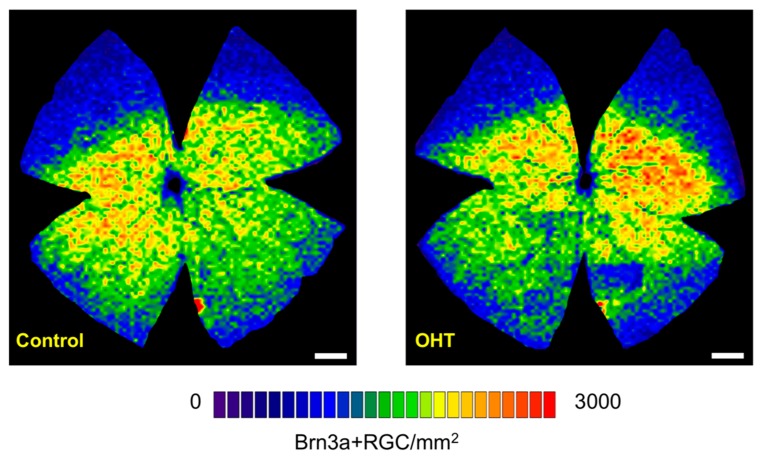
Effect of chronic IOP elevation on Brn3a + RGC isodensity maps. Isodensity maps showing the distribution of Brn3a + RGCs in a control and its contralateral treated retina. There were no significant differences in the total numbers of Brn3a + RGCs (*p* > 0.05) at the 12 week follow-up. This is a representative image of a typical distribution of Brn3a + RGCs across the retina. Brn3a + RGCs are denser in the central and medial retina and scanter in the periphery. Density color scale ranges from 0 (dark blue) to 3000 (red) Brn3a + RGCs /mm2 or higher. The dorsal pole is located at the 12 o’clock position for all retinas. Scale bar = 1 mm.

After the ERG recordings and OCT measurements, the effect of chronic IOP elevation on RGC density was also measured analyzing Brn3a + RGCs flat-mounted retinas. Brn3a + RGCs did not show any significant changes in the hypertensive eyes (91.279 ± 2424 cells/mm^2^) compared with the control fellow eyes (91.606 ± 2.898 cells/mm^2^) at week 12 (t-test, *n* = 4 per group, *p* > 0.05). These hypertensive and fellow reference retinas depicted the typical Brn3a + RGC distribution in the retina (Figure 6), showing higher densities in the visual streak and peak densities in the superior-temporal retina [27,28]. 

**Figure 7:**
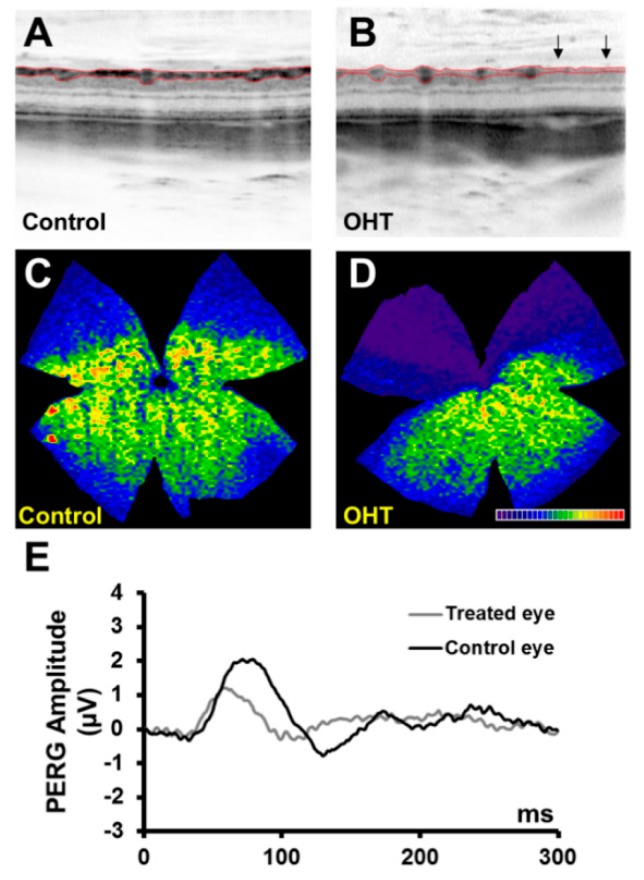
Two hypertensive eyes eventually progressed to develop distinct inner retinal changes. Here, we show a representative animal with a mean IOP in the hypertensive eye of 21.67 mmHg and of 11.70 mmHg in the fellow eye that was shown to develop structural retinal changes 24 weeks post-injection. (**A**,**B**) The RNFL + GCL + IPL showed significant thinning (60.33 µm) in the hypertensive eyes vs. the control eyes (73.44 µm), as measured with the OCT. (**C**,**D**) Isodensity maps depicting the Brn3a + RGC distribution in a hypertensive and control retina. In contrast to the normal distribution of Brn3a + RGCs observed in the control right retina (**C**), the hypertensive retina (**D**) shows a typical sector located approximately between the 9:30 and 1:30 clockface positions that are almost free of Brn3a + RGCs. (**E**) PERG P50 amplitude values were also significantly reduced in the hypertensive eye (1.19 µV) compared to the control eye (2.09 µV).

By week 24, two animals (2/13, 15.3%) showed distinct structural RGC losses that were first spotted with the OCT. In Figure 7, structural and functional data from one of these representative rats are shown. The mean IOP of the hypertensive eye was 21.67 mmHg while in the fellow eye it was 11.70 mmHg. The OCT measurements of the RNFL + GCL + IPL thickness were significantly reduced in the hypertensive eye (60.33 µm) compared to the control fellow eye (73.44 µm), (Figure 7A,B). Furthermore, the Brn3a + RGC isodensity map revealed how the RGC loss was confined preferentially to the superior retina of the hypertensive eye (53.713 cells/mm^2^) compared to the control fellow eye (80.007 cells/mm^2^) (Figure 7C,D). PERG P50 amplitude values were also significantly lowered in the hypertensive eye (1.19 µV) compared to the control eye (2.09 µV) (Figure 7E).

## 4. Discussion 

In this experimental study we detailed a new method of injecting polidocanol, a foamed sclerosant drug, in the aqueous humor outflow pathway to generate moderate and persistent elevated IOP and RGC dysfunction. This model results in a moderate pressure elevation that when sustained will produce functional changes suggestive of optic nerve damage but without obvious morphologic changes until late. The data indicates that the majority of the followed-up rodents that developed initial OHT (17 out of 25 prepared rats) did not show alterations of their physiological responses in their full-field ERG (photoreceptor a-wave and bipolar cell b-wave) or structural (OCT) or cell loss (Brn3a + RGCs) after polidocanol injection in the perilimbal veins. An advantage of this model over other experimental ocular hypertension models, such as the injection of hypertonic saline [6], circumlimbal suture [32], episcleral vein occlusion [8], or lasering of the limbal and perilimbal tissues [2], is the absence of excessively high IOP, anterior chamber inflammation, and outer retina damage. In addition, the model is appropriate for evaluating moderate IOP-related changes that do not quickly lead to actual RGC death and it can be used to test new IOP-lowering drugs in early glaucoma stages more effectively. 

We found that polidocanol injection into the aqueous humor outflow pathway is a straightforward way of producing sustained moderate chronic hypertension in rat eyes. The IOP elevation mechanism probably consisted of induced lack of drainage due to the sclerosant drug. Additionally, aqueous outflow is likely to be affected due to congestion in the aqueous drainage veins. Injections of polidocanol into the episcleral vessels produced a moderate and sustained higher mean IOP (at an average of 21%) for at least nine weeks when compared with the contralateral eye and with no additional re-treatment. 

Rodent models of experimental glaucoma have shown that lower levels of chronically elevated IOP might be more significant to human OHT and primary glaucoma [3]. For low-to-moderate elevations of IOP, as achieved in the present experimental model, we were able to observe RGC function losses as assessed by the PERG well before structural changes were evidenced using RGC isodensity maps and OCT. Our data are consistent with the assumption that RGC dysfunction occurs well before RGC death [33,34]. 

In humans, the rate of conversion of untreated OHT to glaucoma has been reported to be around 1% per year [35]. In our study, as the aggregated sustained injury (mean IOP) increased, RGC function anomalies became apparent, as did signs of microstructural damage to inner retinal layers in some of our experimental animals. PERG has been shown to be significantly abnormal in human subjects with chronic OHT and glaucoma [36,37,38,39,40,41]. It has also been reported that the PERG loss does not correlate with the OCT RNFL thickness changes [42]. This evidence backs the suggestion that RGC dysfunction may anticipate axonal and cell loss [13,31,43,44].

The association of nerve damage and IOP suggests that the RGC damage is, in general, related to the duration and degree of IOP elevation, although there may be some individual variation in IOP susceptibility [5]. Previous rodent models of glaucoma have shown an association between IOP elevation and RGCs loss [45], although only a few studies have reported preferential RGC dysfunction [5,31,46]. 

PERG can be very useful to demonstrate early inner retina function loss in rodent models of chronic IOP elevation [47], well before any structural damage of the inner retina is evident. Our results showed polidocanol injection induced a significant loss of the inner retinal function (P50) in rats that correlated well with the degree of IOP elevation. There is evidence that elevated IOP in glaucoma rodent models does significantly lower light-evoked spike responses of ON and OFF RGCs before any documented structural damage and that this reduction is mainly provoked by the ON cone bipolar cell signals’ lower sensitivity to the ON RGCs and by the amacrine cell signals’ loss of sensitivity to the OFF RGCs [33]. 

Currently, PERG is the most specific technique for electrophysiological evaluation of RGC function in rodent glaucoma models. However, the function of RGCs can also be non-invasively assessed by using different forms of the flash ERG technique that highlight the activity of inner retina neurons, e.g., positive scotopic threshold response (pSTR), negative scotopic threshold response, (nSTR), photopic negative response (PhNR), and oscillatory potentials. It is not yet well known which of these inner-retina-sensitive ERG components could be most sensitive to glaucomatous damage and the choice of one electroretinographic technique over the other is based on the evaluation of the advantages and disadvantages on each particular experimental model [40,48].

One of the main drawbacks with most ocular hypertensive models is that the outer retina is frequently damaged due to excessive IOP values, as shown by the ERG and morphological studies [2,46,49,50,51]. However, in the present model, no significant changes were ever detected in the outer retina responses as recorded in the ERG.

Up to one third of the animals did not respond to a single injection of polidocanol, so there are several limitations to the use of this model in rodents. First, the surgical technique of inserting a microneedle into the rat episcleral vein is difficult and requires significant training. Second, IOP was measured under isoflurane anesthesia, and this is known to lower IOP, thus the moderate elevations in IOP observed may in fact be larger than expected. Third, irregular obstruction of the outflow pathway can result in a large variation in subsequent IOP elevation. Histology of non-responsive eyes was not done, so one may assume that the lack of responsiveness could be attributed to a technical issue, being a technically challenging procedure and the polidocanol did not reach the outflow tissues or, on the contrary, if polidocanol reached the outflow tissues it did not induce enough IOP elevation. 

In summary, a selective loss was detected in the PERG at low levels of chronically elevated IOP before any inner retina structural changes were detected. The magnitude of IOP elevation in this model is lower than in other experimental rodent OHT models, with late damage occurring to the inner retina while the outer retina remains intact. Therefore, this ocular hypertensive model can facilitate experimental studies at early stages of glaucoma when OHT could potentially be treated.

## Abbreviations

OHT	Chronic ocular hypertension
IOP	Intraocular pressure
RGC	Retinal ganglion cells
PERG	Pattern electroretinogram
ERG	Full-field electroretinogram
RNFL	Retinal nerve fiber layer
OCT	Optical coherence tomography
